# Models of Care in Providing Comprehensive Healthcare on Cancer Survivors: A Scoping Review with a TIDieR Checklist Analysis

**DOI:** 10.3390/ijerph21020122

**Published:** 2024-01-23

**Authors:** Martina Torreggiani, Deborah Maselli, Stefania Costi, Monica Guberti

**Affiliations:** 1 Health Professions Department, Azienda USL-IRCCS of Reggio Emilia, 42123 Reggio Emilia, Italy; 2 International Doctorate School in Clinical and Experimental Medicine, Università degli Studi di Modena e Reggio Emilia, 41125 Reggio Emilia, Italy; deborah.maselli@unimore.it; 3 Physical Medicine and Rehabilitation Unit, Azienda USL-IRCCS di Reggio Emilia, 42123 Reggio Emilia, Italy; stefania.costi@unimore.it; 4 Research and EBP Unit, Health Professions Department, Azienda USL-IRCCS of Reggio Emilia, 42123 Reggio Emilia, Italy; monica.guberti@ausl.re.it

**Keywords:** cancer survivors, healthcare service, model of care, oncology service, survivorship

## Abstract

Background: The study’s aim is to identify the models of care used to provide survivorship care plans (SCPs) to cancer survivors in healthcare services, describing what kind of professionals are involved, in which settings and timings, and their feasibility. Methods: The Joanna Briggs Institute methodology for scoping reviews is followed. Studies that considered the SCPs applying different models of care, in any healthcare setting on any adult cancer survivors who completed oncological treatments, have been included. Pubmed, Embase, Cochrane Library, Scopus, and Cinahal were searched from 2013 to 2023 with these keywords: “Survivorship Care Plan”, “Oncology”, and “Program”. The study selection process was reported with the PRISMA-ScR. A total of 325 records were identified, 42 were screened, and, ultimately, 23 articles were included. Results: The models of care include: SCP standardization in hospitals; self-support oriented; consultation-based; primary or specialist direct referral; shared care; a multimodal approach. Multidisciplinary teams were involved in the SCP models of care. The settings were private clinics or cancer centers. One-hour SCP interventions were most frequently delivered through in-person visits, by telephone, or online. Conclusions: Implementing SCPs is feasible in healthcare contexts, but with challenges, like time and resource management. Patient-centered programs promoting coordinated care are promising models of care.

## 1. Introduction

Globally, cancer is the second leading cause of death, with 9.6 million deaths in 2018 [1]. In countries where health systems are strong, the survival rates of many types of cancers are improving thanks to accessible early detection, treatments, and survivorship care [1]. Furthermore, recent oncological clinical research is integrating long-term follow-up and health-related quality of life [2]. The National Cancer Institute’s Office of Cancer Survivorship (NCCS) defines cancer survivorship as the life experience of a person with cancer after treatment until the end of life. It stresses how discussing survival problems with carers can give hope to newly diagnosed patients and support them in being an active part of their treatment path [3]. However, the health needs associated with cancer survivorship are often complex and require specific and personalized approaches [4]. Moreover, these needs evolve along with scientific progress, as well as in survivorship policies and programs, that must also face context-related issues to guarantee equal access to quality care [5]. Survivorship care plans (SCPs) empower care coordination, optimizing follow-up care with various providers [6]. SCPs provide guidelines and support cancer survivors (CSs) in promoting healthy behaviors and tailored lifestyle recommendations, increasing their knowledge on diagnosis, the late effects of cancer treatments, and recurrence [6]. SCPs appear feasible, but additional research is needed to clarify their effectiveness and implementation issues [7]. Still, no absolutely replicable reference exists for any well-being environment to reproduce an SCP. All models should provide personalized and comprehensive patient care that meets the long-term individual needs to improve these patients’ overall health and outcomes [8]. In the Italian epidemiological and demographic context, SCP implementation is still sporadic [9]. This scoping review aims to analyze the organizational models of survivorship care in healthcare contexts.

## 2. Materials and Methods

The proposed scoping review was conducted according to the methodology of the Joanna Briggs Institute (JBI) for scoping reviews [10]. The review question was built following the population–concept–context (PCC) framework [10] to ensure clarity. The primary outcome was to identify and describe the models of care used to provide SCPs (concept) to CSs (population) in healthcare services (context). The secondary outcome was to evaluate (1) what kind of professionals are involved in providing SCPs; (2) which settings and timings are planned in the SCPs offered to the patients; (3) the feasibility of the implementation of the SCPs’ models of care. The protocol of this scoping review is registered on Open Science Framework (osf.io/5wm6g; accessed on 22 January 2024).

### 2.1. Eligibility Criteria

The inclusion criteria are as follows: (1) studies that considered the SCP intervention applying different models of care in any healthcare setting; (2) studies that evaluated any outcome on adult CSs that completed the active chemo/radiotherapy treatment or healthcare personnel perceptions; (3) experimental, quasiexperimental, observational study designs, qualitative and mixed-method studies, and also systematic reviews that met the inclusion criteria; (4) studies published from 2013 to 2023. Thus, the exclusion criteria are listed as follows: (I) studies that do not refer to the oncological field; (II) the nonadult population; (III) incomplete or unpublished literature; ineligible evidence types (conference papers, clinical cases, and theoretical/position papers).

### 2.2. Search Strategy

We initially searched for articles in MEDLINE. We evaluated the available evidence in the last ten years to include relevant international experiences of SCP implementation, as their relevance is not only influenced by the time-related scientific progress, but also by the healthcare-context-related issues and by its population. The databases (Pubmed, Embase, Cochrane Library, Scopus, and Cinahl) were searched in May 2023, with no language limitations, with these keywords: “Survivorship Care Plan”, “Oncology”, and “Program”. The search strategy, including all keywords and index terms, was reconciled in each database until content-related saturation. The reference list of all included sources of evidence was screened for additional studies.

### 2.3. Study Selection

**Figure 1:**
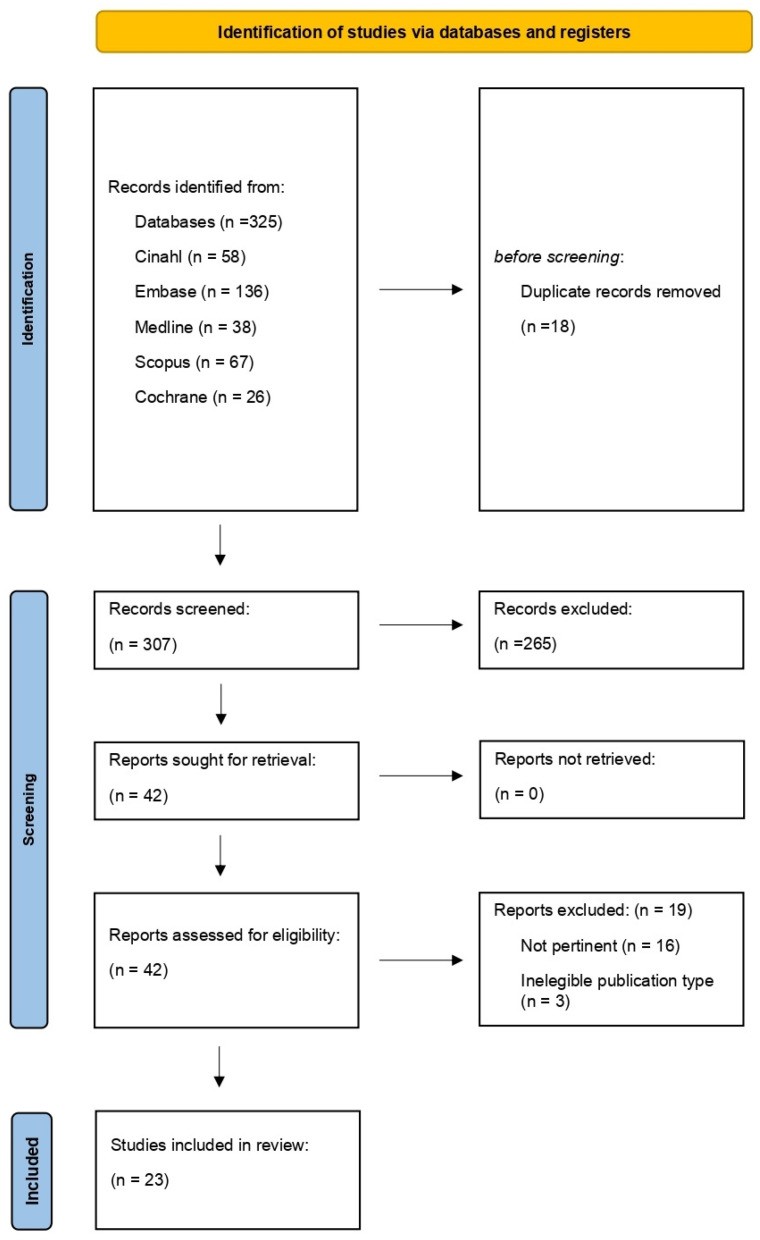
PRISMA flow diagram for the scoping review process. After the title and abstract screening, we assessed 42 studies for eligibility from the full-text analysis. A total of 23 articles met the inclusion criteria and were included in the review.

The study selection process, represented in Figure 1, is conferred in a Preferred Reporting Items for Systematic Reviews and Meta-analyses extension for a scoping review (PRISMA-ScR) flow diagram [10]. Mendeley software (2.92.0.55 version), currently used in the literature [11], was chosen to import the results and remove duplicates. Records were screened for eligibility by two independent reviewers in each phase: firstly, by evaluating the title and the abstract; secondly, after the full-text reading. Any disagreements were discussed at each stage of the selection process by the reviewers. Reasons for the exclusion of studies were recorded and reported.

### 2.4. Data Extraction

The selected articles were extracted into Excel by one reviewer and independently cross-checked. If the full texts were unavailable, the abstracts were considered in the analysis, reported, and discussed separately. Extracted data included the author, year, country, study design, professionals, setting, timing, endpoints, and main results. These elements were sought according to the specific outcomes stated before. A narrative and thematic synthesis was conducted by one reviewer to summarize the results, evaluating the SCP interventions.

### 2.5. TIDieR Checklist

The Template for Intervention Description and Replication (TIDieR) [12], a tool to improve the quality of the reporting and the reproducibility of healthcare interventions, has been used to analyze the SCPs in detail. This checklist is currently used in similar research [13,14]. It consists of a checklist of 12 items and the relative guide, explaining and elaborating each item and examples of appropriate reporting. For complex interventions, as in this case, this level of detail is necessary for each intervention component. However, this information needs to be included or reported.

### 2.6. Critical Appraisal of the Evidence

Although this step is not considered mandatory in the methodology followed, we decided to critically appraise the literature included because it provides more certainty when referring to this intervention, specifically on how it should be methodologically evaluated and implemented in different healthcare contexts. The quality assessment was conducted with the Effective Public Health Practice Project (EPHPP) [15]. It is a widely used instrument with an excellent degree of inter-rating reliability for experimental and observational studies [15]. The score’s sum of the six domains (selection bias, study design, confounders, blinding, data collection methods, withdrawals, and drop-outs) constituted the overall quality rating that could be “strong”, “moderate”, or “weak”. Qualitative studies have been evaluated with the Critical Appraisal Skills Programme (CASP) checklist [16], a commonly used tool in similar research [17,18]. As reported in the recent literature [19], guidelines were assessed with the Appraisal of Guidelines, Research, and Evaluation (AGREE) II checklist [20]. Two independent reviewers conducted the quality assessment and discussed and solved any discrepancies.

## 3. Results

### 3.1. Literature Search Results

As represented in Figure 1, the literature search identified 325 records: 38 from Pubmed, 136 from Embase, 26 from the Cochrane Library, 67 from Scopus, and 58 from Cinahl. The results obtained were relevant and satisfactory. As many duplicates were retrieved, other databases (e.g., Web of Science and Google Scholar) were not consulted further. After deleting 18 duplicates, 307 articles were screened by title and abstract reading. A total of 42 papers were assessed for eligibility: 19 studies were of the excluded: not pertinent (N = 16) and not eligible publication types (N = 3). Finally, 23 articles were included in the review. As almost half of the evidence included was older than five years, we agreed to present them separately into two subgroups (before and after 2019): this permitted a more specific analysis considering the most recent technoscientific progress. The main findings are summarized in Appendix A and Appendix B. The SCP interventions of the studies included are analyzed with the TIDieR checklist in Appendix C and Appendix D.

### 3.2. Characteristics of the Included Literature

The articles involved participants from America (n = 17), Asia (n = 2), and Oceania (n = 4). The study designs were observational (n = 7), experimental (N = 12), mixed-methods (N = 2), qualitative (n = 1), and guideline (n = 1). Specifically, the experimental studies were: randomized controlled trials (RCTs) (N = 4), pilot studies (N = 4), implementation projects (N = 2), and quality-improvement projects (N = 2). Thirteen studies evaluated an SCP intervention in clinical contexts [21,22,23,24,25,26,27,28,29,30,31,32]; six studies described the tool-development or integration processes [33,34,35,36,37,38]. The SCPs were confronted with usual care in four RCTs [39,40,41,42]. One guideline on breast cancer survivors (BCSs) was found [43]. BCSs were involved in twelve studies [24,27,29,31,32,33,34,36,37,40,41,43]; six with various types of cancer [21,22,23,28,30,38]; one with the bladder cancer type [26]; two with colorectal cancer (CRC) types [23,24]; one with the lung cancer type [35]; one with the melanoma type [25]; one with the head–neck cancer type [39]; one with the gynecological cancer type [32].

### 3.3. Critical Appraisal with the Sources of Evidence

As chronologically reported in Table 1, the quality of the evidence was variable: strong (N = 4), moderate (N = 11), and weak (N = 6). The study design, the robustness of the methodology, and the sample size were the main elements influencing the rates. The overall quality of the guideline [43] was 6/7 on the AGREE II scale. The qualitative study [31] was considered of good quality from the CASP checklist.

### 3.4. Results of the Individual Sources of Evidence

We present the findings of studies published before 2019. The objectives of risk-adapted visits (RAVs) in the Living in the Future (LIFE) program described in Rosenberg et al. [21] are focused on patient reintegration into primary care and community resources, and educating CSs on diagnosis, treatments, and recommendations for preventive healthcare. The education addresses lifestyle nutrition/fitness, genetics, sexuality, employment, cognition, and lymphedema. Rosales et al. [22] implemented a successful survivorship model in a tumor institute: they reviewed 118 medical records to evaluate survivorship needs and satisfaction. Weight management (35%), fatigue (30%), and sexuality (27%) resulted in some of the most frequent worries. This SCP improved patient engagement, satisfaction, and care coordination. Jefford et al. [23] developed an analysis framework of reports to synthesize the key themes, enablers, and challenges of six 2-year Victorian Cancer Survivorship Program (VCSP) projects. The interventions were considered appropriate by CSs. Strong leadership, workforce education, risk-stratified pathways, and shared personalized care models were the primary enablers. The lack of tool validity, the limited evidence, the workforce redesign, and issues around survivorship terminology were challenging factors. Then, Jefford et al. [42] conducted a multicenter RCT aiming to improve the quality of life (QOL), psychological concerns, and CRC survivors’ care needs (SCNs). The intervention, including the educational materials, needs assessment, end-of-treatment session, and three follow-up telephone calls, was compared to the usual care (UC) (N = 110). Between-group differences in distress, SCNs, and QOL at 2 and 6 months were small and nonsignificant. The SCP group was more satisfied with the SCP than the UC group. O’Hea et al. [37] presented the development results of a web-based breast cancer SCP: the Polaris Oncology Survivorship Transition (POST) matched data from the electronic health records (EHRs) and oncology care providers (OCPs). Twenty-five women ending treatment in the past year were selected from outpatient clinics and chemotherapy units. They received the POST computerized assessment and a tailored SCP. A total of 70% of the responders rated the SCP highly satisfactory. Berman et al. [35] used the OncoLife and the LIVESTRONG care plans to identify demographic, treatment, and toxicity data of primary lung cancer survivors: of 689 patients, neurocognitive adverse effects were the most frequent (48.8%). Dulko et al. [24] aimed to evaluate the process of an SCP completion (patient response rate: 73%), and to survey oncology staff (OF) (response rate: 94%) and primary care physicians (PCPs) (response rate: 71%) regarding the challenges of SCPs in two facilities. Despite its usefulness, the creation time and insufficient knowledge of CS issues were perceived barriers. Grant et al. [34] described the implementation of follow-up models for BC survivors across 14 Canadian Regional Cancer Centers: all regions used SCPs and patient education materials, direct-to-primary care, transition clinics, and shared respect. A total of 85% of patients reported feeling adequately prepared for the transition to primary care. Downs-Holmes et al. [33] described the necessary steps for developing and implementing an institution-specific survivorship program to fulfill the new standards for survivorship care. Patt et al. [28] implemented an SCP in a cancer center, creating a toolkit, interorganizational collaboration, and assembling a working team. In Runowicz et al. [43], the purpose of the guideline was to provide recommendations for breast cancer survivors’ care: recommendations concerned surveillance for breast cancer recurrence, long-term effects, health promotion, and care coordination. Tevaarwerk et al. [36] described a clinical trial assessing the survivors’ knowledge after the receipt of treatment summaries. The exploratory analysis showed that a significant proportion of treatment summaries contained at least one error (25%) or omission (22%).

The findings of the studies published after 2019 are presented below. Williamson-Butler et al. [40] aimed to compare an RCT design with an SCP program (POST) to UC: the outcome was set on the quality of discussion (QOD) between providers and patients. At their last treatment visit, two hundred patients were randomized. The POST women reported a better QOD. Su et al. [41] employed an RCT to search if breast CSs receiving a web-based SCP were more likely to improve on at least one of the four targeted issues than the attention controls. A total of 70.9% of women improved in fertility-related concerns, hot flashes, vaginal symptoms, and contraception compared to 57.3% of the control group. Lee C.T. et al. [26] described, with a mixed-methods approach, the feasibility of SCPs among breast cancer survivors. Patients found high acceptability and engagement; 59 SCPs were completed by providers without any difficulty, confirming the clearness, relevance, and feasibility. Lai-Kwon et al. [25] assessed the feasibility and acceptability of a nurse-led, telehealth-delivered SCP for metastatic melanoma survivors. The participation rate was 57%; 97% completed the program, demonstrating its utility and acceptability (3/4 AIM items). Ivanics et al. [27] conducted a project evaluating two SCP programs implemented in a cancer institute with a quality-improvement Plan–Do–Study–Act model. System II (treatment summaries by multidisciplinary breast specialists) had fewer inaccuracies than System I (treatment summaries by nonspecialist breast clinic staff). Lee L-Y. et al. [39] employed an RCT design to evaluate the effects of a nurse-led SCP compared to usual care on emotional distress, physical and mental health, social support, and resilience among 100 dyads (caregivers and patients with advanced head and neck cancer). In the nurse-led SCP, the outcomes slightly improved in six months, with statistical significance. Napoles et al. [29] evaluated the feasibility and acceptability of a linguistically suitable Spanish-speaking breast cancer survivor SCP. A total of 83% of women completed all five coaching calls. A total of 81% rated the app’s overall quality as “very good” or “excellent.” McGrath et al. [38] standardized and integrated an SCP into the EHR: this increased the participation of other specialists and the rate of completion from 10% to 34%. Glaser et al. [32] described an SCP in a survivorship clinic with a network of support services. A total of 908 CSs received the SCP and personalized complementary care. Corsini et al. [30] reported a multicentric pilot study that tested SCP tools with a quality-improvement approach: based on the 43 consultations made, barriers included perceived knowledge, the time to complete the documentation, referral pathways, and the lack of administrative support. Fok et al. [31] explored the perspectives of PCPs towards managing BCSs in shared-care with oncologists. Most PCPs referred to limitations in managing acute and nononcological issues. PCPs’ role may grow, including cancer surveillance and unmet needs.

### 3.5. Synthesis of the Results

#### 3.5.1. Professionals Involved in Providing the SCP

Generally, the SCP approach is multidisciplinary. Although the professionals most frequently involved in the SCPs are medical oncologists (MOs), PCPs, and nurses, in six studies, the MOs/PCPs and the nurses worked together [21,26,29,30,35,37]; in two studies, only MOs/PCPs were involved [31,41]. A sizeable multidisciplinary approach was described in four studies [23,28,33,34]. In seven studies, the SCP was provided by a nurse [22,24,25,36,39,40,42], and in two studies [30,38], they worked in teams. Two studies referred to the OS [27,32]. There were no substantial differences between the professionals involved in the SCPs before or after 2019. This suggests that the results were not distinctly susceptible to scientific progress over this period.

#### 3.5.2. Setting and Duration of the SCP Interventions

Eleven studies were multicentric [21,22,23,24,28,30,34,36,37,39,42]. Eleven studies involved one center [25,26,27,31,32,33,35,38,40,41]. Four studies enrolled patients from outpatient private clinics [22,26,27,37]. Cancer centers/survivorship clinics were involved in nine studies [28,30,31,32,33,34,36,38,39]. Tevaarwerk et al. [36] provided intervention with an in-person visit or by telephone, while, in seven studies, intervention was provided only by an in-person visit [22,24,26,28,30,37,38]; in three studies, intervention was provided only by telephone [25] or was web-based [35,41]. Five studies planned remote follow-up after the first visit [21,27,39,40,42]. One study organized home visits [29]. Jefford et al. [23] analyzed various models:

- shared care with discharge to a general practitioner (GP), with one or two appointments supporting health promotion;
- specialist care with GP support through multidisciplinary visits;
- self-support or community services referral.

According to Glaser et al. [32], the survivorship clinic offers a one-time visit with referrals, transition to long-term care, or self-referral. Most studies that reported this information provided a one-hour intervention (N = 6) [21,22,25,28,39,42]; two studies [27,40] provided an intervention that lasted less than one hour; the other two studies provided an intervention that lasted more than one hour [22,30]. Studies conducted before 2019 were mainly multicentric. Meanwhile, the most recent studies were developed in a single health center. Specialized cancer centers and private clinics had the same commitment over several years in the proposals of the survivorship care plan.

#### 3.5.3. Models of Care

As describing complex interventions can be challenging, the TIDieR checklist helped us to summarize the core elements of the different experiences of providing survivorship care and how they are provided in the healthcare contexts:

- SCP standardization in hospital care: the SCP was used to collect information about specific survivorship issues [35] or for quality-improvement projects aiming to (1) improve the efficacy with the EHR integration of the SCP document [38,40]; (2) improve the accuracy of the SCP document [36]; (3) improve the knowledge and consciousness about specific survivorship issues [41]; (4) improve the complete comprehension of the SCP document [29].
- Self-support oriented [23,41].
- Consultation-based: several studies reported similar experiences. The SCP intervention was created and completed with the patient during a hospital visit. The SCP document was recorded within the patient chart, and a summary of the indications were given to the patient and sent to the PCP. Clinicians managed the follow-up remotely [22,24,26,28,30,39,42]. Two studies reported a project where a specific computer program was built [37] and used [40]: this integrated information between the EHR, MO, and patients, and was used to create the SCP. One study organized telehealth consultations [25]. In two studies, the ON managed the intervention [21,36].

Most of the experiences reviewed involved multiple stakeholders but varied in the focus of the care provider referral:

- PCP direct referral [34].
- Specialist direct referral [23].
- Integration between specialist and primary care: the key elements of the integration were multimodal resources, dedicated clinics, and a shared-care model. One study used different resources (visits, summaries, and a phoneline) to catalyze the passage from active treatment to follow-up care [21]. Glaser et al. [32] reported the experience of a survivorship clinic linked with external services concerning wellness and nutrition. A transition clinic within one of the cancer centers involved in the study of Grant et al. helped transition survivors back to their PCPs [34]. A shared-care model was applied in coresponsibility between the MO and PCP [34], as in Fok et al. [31], where the approach was risk-stratified. In Jefford et al. [23], the patient was discharged to the PCP, with one or two survivorship appointments supporting health promotion.
- Multimodal approach: the studies [23,30,34] reported different experiences in testing the models of care that varied according to the service available in the territory.

Studies showed similarities in adapting the models of care to each context over time, specifically on accessibility and follow-up. However, digital and innovative strategies have grown in the last five years.

## 4. Discussion

A scoping review methodology was selected [10], as it provides literature mapping on a specific topic, showing concepts, evidence gaps, and the types of available studies [44]. The overall body of evidence concerning the topic was variable and of moderate quality. Breast cancer was the most frequent when a specific type of cancer was analyzed. The SCPs were confronted with usual care in the RCT designs: one found statistically significant differences in the SCP group on social support, emotional distress, physical and mental health, and resilience improvement after six months [39]. Nevertheless, a similar study [42] conducted in 2015 with a bigger sample size found no significant differences other than a higher satisfaction rate. A significantly higher quality of discussion with the providers was found in the SCP group of another study [40].

The SCPs involved are primarily provided in multidisciplinary, patient-centered contexts, integrated with primary care at different levels. The results were valid in promoting individualized healthcare, self-management, and well-being. Implementing and adapting an SCP in different healthcare contexts appears to be a feasible intervention. Many human and technological resources provide the intervention presented in the reviewed studies, negatively impacting costs [21,34]. Most emerging challenges, like the lack of time, administrative support, and specific training, worsen as the medical facility has less volume of patients and a greater distance to the central hub [30]. Nevertheless, innovative models of care that consider community characteristics and needs (self-support and service-oriented, web-based interventions, and periodic home visits) [25,36,41] are promising in optimal timing, resourcing, and cost-effectiveness, and can be studied with robust design studies. Technological integration in usual care and the balance between specialty and primary care remain challenging in many contexts. The main enablers in implementing SCPs were focused on preparing survivors for post-treatment care. Some survivors face new models of survival assistance immediately after treatment. In contrast, others need time to process and recognize the end of treatment before contemplating survival challenges. Stratified pathways of early preparation for survival are based on individual needs and self-management with shared-care models [21]. Nursing survival models have successfully provided comprehensive care, addressed the unique needs of CSs, and improved patient self-management [23,25,26,33]. The introduction of survival programs increases the awareness among health professionals of the need to enhance post-treatment care. The limited capacity of outpatient services has led to the development of new approaches to survival care, such as the passage to primary care settings [31,41]. Clinical solid leadership, community organizations, stakeholders, and primary care providers are crucial to successful implementation [23,28,33,34]. In this regard, training and education are also determinants to support professionals in involving patients and family members appropriately. A barrier to the successful implementation of SCPs is the lack of valuable assessment tools to predict the needs of survivors [21,33,37]: the documentation and follow-up of survival care, including goal setting, symptom reassessment, and follow-up, are often inadequate, which may hinder the provision of comprehensive care [24]. The literature needs more evidence to support new care models. Another relevant aspect is redesigning the health workforce, as providing survival care requires specific education and training for operators in different care settings [29,31]. In addition, the care teams needed help identifying patients who had finished the therapy and were suitable for the SCP due to the incomplete treatment plans or the unclear responsibility of the SCP among different specialties [24]. Moreover, one of the main obstacles to evolving the SCP is linked to time: the study of medical records, especially for patients who have received treatment in different settings, where obtaining prior or external data, including chemotherapy data, has been reported as challenging. The difficulty in updating SCPs appears familiar to many care plans, and there have been mixed responses as to when the SCPs should be updated for disease progression or new primary diagnoses. Another impact is the limited access to survivors and insufficient knowledge, where PCPs have reported limited access to survivors and inadequate understanding of cancer survivors’ problems, indicated as obstacles to the follow-up organization. The barriers highlighted the need for institutional support, better communication between healthcare providers, and potential improvements in EHR systems to facilitate the implementation of the SCPs [25,29,35,38,41]. Being able to take advantage of technological advancements to meet the needs of survival could provide the assurance of equal access to care plans, ensuring their traceability and redirection to potential health benefits, and, consequently, with medical management for the long-term results of cancer survival.

### 4.1. Limitations

The present study has some limitations. A small number (N = 23) of studies were eligible for this review; almost half of the studies were dated beyond 5 years. The small sample sizes of several of the non-RCT studies analyzed could not permit the generalization of the results. Moreover, European experiences needed to be included in the literature examined, making it difficult to compare, and eventually adapt, the results in universal healthcare systems.

### 4.2. Implication for Practice

Different SCP models are potentially replicable in different healthcare contexts, like multidisciplinary nurse-led transition education programs. The studies demonstrate that nurses are well-positioned to provide patient education and support regarding SCPs, helping patients understand their health conditions, treatments, and preventive healthcare [25]. Many studies have shown that nurse-managed survival care is safe, comprehensive, and successful when placed in the healthcare coordination of cancer patients [25,30,32,38,39,40], even with digital health [29]. The nursing profession is indispensable in health education to achieve the physical and psychosocial results of the patient. The other fundamental aspect is related to the economic benefits derived by the time savings and the reduced use of health resources compared to the care led by specialists. They can also empower cancer survivors to take an active role in their wellness and connect them to community resources for emotional and physical recovery [26] as facilitators of the transition from oncology care to primary care. Further research may focus on innovative and cost-effective models of care combined with solid competence and training, decisive in assuring sustainable and high-quality SCPs.

## 5. Conclusions

This scoping review aimed to identify the models of care used to provide survivorship care plans (SCPs) to cancer survivors in healthcare services, describing what kind of professionals are involved, in which settings and timings, and their feasibility. The main models of care implemented to provide SCPs are standardization in hospitals, self-support oriented, and consultation-based, with primary or specialist direct referral, with a shared-care or a multimodal approach. Multidisciplinary teams are mostly involved in private clinics or cancer centers. One-hour SCP interventions were most frequently delivered through in-person visits or digital health. Implementing SCPs is feasible in healthcare contexts, but with challenges, like time and resource management. Patient-centered programs promoting coordinated care are promising models of care. Promoting dynamic care between the hospital and territory may ensure a more graded assistance: nurses are promising in this vision, favoring continuity, quality, and the appropriateness of survivorship care.

## Figures and Tables

**Table 1:** Quality assessment of the included quantitative studies with the Effective Public Health Practice Project (EPHPP).

Quantitative Study	EPHPP
Lee et al. (2023) [39]	Strong
Lai-Kwon et al. (2022) [25]	Moderate
Williamson-Butler et al. (2022) [40]	Strong
Lee et al. (2020) [26]	Moderate
Corsini et al. (2020) [30]	Moderate
Glaser et al. (2019) [32]	Moderate
McGrath et al. (2019) [38]	Weak
Su et al. (2019) [41]	Strong
Nàpoles et al. (2019) [29]	Moderate
Ivanics et al. (2019) [27]	Moderate
Tevaarwerk (2017) [36]	Moderate
Rosenberg et al. (2016) [21]	Moderate
Jefford et al. (2016) [42]	Strong
O’Hea et al. (2016) [37]	Moderate
Berman et al. (2016) [35]	Weak
Grant et al. (2015) [34]	Weak
Jefford et al. (2015) [23]	Moderate
Rosales et al. (2014) [22]	Weak
Downs-Holmes et al. (2014) [33]	Weak
Patt et al. (2013) [28]	Weak
Dulko et al. (2013) [24]	Moderate

## Data Availability

No new data were created or analyzed in this study. Data sharing is not applicable to this article.

## Appendix A

**Table A1:** Literature results from 2013 to 2018.

Author Year	Country	Setting	Professionals	Type of Study	Population	Endpoint	Results
Rosenberg et al. (2016) [21]	America	Hospitals	Nurse and MO	Observational study	Different types of cancer	Evaluating RAVs in promoting individualized healthcare and self-management during survivorship transition.	A total of 1615 questionnaires were completed. For the strongly agree/agree ratings, 94% felt more confident in communicating their treatment issues to other healthcare providers; 90% felt more comfortable recognizing symptoms to report; 98% had a better appreciation for community programs.
Rosales et al. (2014) [22]	America	Outpatient private clinics	Nurse	Observational study	Different types of cancer	Implementing a successful SCP.	For a total of 118 medical record reviews and follow-up telephone calls, the concerns were weight management (35%), fatigue (30%), sexuality (27%), anxiety (23%), caregiver stress (17%), and depression (16%).
Jefford et al. (2015) [23]	Oceania	Hospitals and primary care	Multidisciplinary	Pilot study	Different types of cancer	Synthesizing key themes, enablers, and challenges about six 2-year projects of the VCSP.	The lack of tool validity, the limited evidence, the workforce redesign, and the issues around the SCP terminology were challenging factors.
Dulko et al. (2013) [24]	America	Hospitals	Nurse	Observational study	Breast and colorectal cancer	Evaluating the SCP completion and surveying oncology staff and PCPs regarding the challenges of implementing the SCP.	The patient response rate: 73%. The oncology staff response rate: 94%. The PCP response rate: 71%. Creation time may be a barrier to SCP implementation. CSs find SCPs useful, but PCPs had insufficient knowledge of the CS issues. Incorporating SCPs in EHR may facilitate the SCP implementation.
Patt et al. (2013) [28]	America	Cancer centers	Multidisciplinary	Observational study	Different types of cancer	Implementing an SCP within a suburban oncology practice.	Offering these services to patients in their communities means that we will provide a higher quality of care and help them.
Downs-Holmes et al. (2014) [33]	America	Cancer center	Multidisciplinary	Implementation projects	Breast cancer	Describing the steps for the development and implementation of an institution-specific SCP to fulfill the new standards for SCPs.	The steps provided within the context can be adapted to any cancer type with minor modifications.
Grant et al. (2015) [34]	America	Cancer centers	Multidisciplinary	Observational study	Breast cancer	Implementation of sustainable models of follow-up care across 14 Canadian Regional Cancer Centers.	All regions used the SCP and patient education materials, direct-to-primary care, transition clinics, and shared care. A total of 85% of the 752 patients reported that they felt prepared for the passage to primary care.
Berman et al. (2016) [35]	America	Tele-health	Nurses and PCPs	Observational study	Lung cancer	Generating SCPs using patient-reported outcomes and web-based programs.	Neurocognitive adverse effects (e.g., fatigue and cognitive changes) were the most common (48.8%), especially among those receiving chemotherapy.
Tevaarwerk et al. (2017) [36]	America	Cancer centers	Nurse	Observational study	Breast cancer	Describing the change in survivor knowledge after the receipt of TSs delivered as part of the SCPs.	A significant proportion of SCPs prepared for CSs enrolled in a clinical trial contained at least one error (25%) or omission (22%).
O’Hea et al. (2016) [37]	America	Outpatient private clinics	Nurses and MOs	Pilot study	Breast cancer	Development and field test of a web-based BC SCP system.	The POST computerized assessment and a tailored SCP were provided for 25 women ending treatment in the past year. A total of 70% of the 23 responders rated the SCP as satisfactory.
Jefford et al. (2016) [42]	Oceania	Hospitals	Nurse	RCT	Colorectal cancer	SCP + UC (N = 107) vs. UC (N = 110) to improve psychological distress, SCNs, and the QOL of patients with CRC.	Between-group differences in the SCNs and the QOL at 2 and 6 months were small and nonsignificant. Patients in the SCP group were more satisfied with the SCP than those in the UC group.
Runowicz et al. (2016) [43]	America	NA	NA	Guideline	Breast cancer	The purpose of the ACS/ASCO BC SCP was to provide recommendations to assist clinicians in the care of female adult survivors.	Surveillance for BC recurrence, screening for second primary cancers, the assessment and management of the physical and psychosocial LLTEs of BC and its treatment, health promotion, and care coordination.

PCPs = primary care physicians; RCT = randomized controlled trial; CRC = colorectal cancer; SCP = survivorship care plan; VCSP = Victorian Cancer Survivorship Program; SC = survivor care; SCNs = supportive care needs; QOL = quality of life; CS = cancer survivorship; SCCP = survivorship care planning program; RAVs = risk-adapted visits; LIFE = Living in the Future; POST = Polaris Oncology Survivorship Transition; EHR = electronic health record; OCPs = oncology care providers; ACS = American Cancer Society; ASCO = American Society of Clinical Oncology; LLTE = late/long-term effect; TS = treatment summaries; EMR = electronic medical record; IG = intervention group; CG = control group; MO = medical oncologist; BCS = breast cancer survival; UC = usual care; QOD = quality of discussion; NA = not applicable.

## Appendix B

**Table A2:** Literature results from 2019 to 2023.

Author Year	Country	Setting	Professionals	Type of Study	Population	Endpoint	Results
Lai- Kwon et al. (2022) [25]	Oceania	Tele-health	Nurse	Pilot study	Metastatic melanoma	Verification of the feasibility, acceptability, and utility of a novel model of nurse-led, telehealth-delivered SCP.	The participation rate was 57%; 97% completed the program, demonstrating its utility and acceptability.
Lee C.T. et al. (2020) [26]	America	Outpatient private clinic	Nurse and MO	Mixed-methods	Bladder cancer	Acceptability and feasibility of a BC-specific SCP.	A total of 59 SCPs were completed by the providers. Clinical resources were required to ensure the appropriate implementation of the BC SCPs.
Ivanics et al. (2019) [27]	America	Outpatient private clinic	Oncological staff	Pilot study	Breast cancer	Evaluating two different SCP programs implemented with a quality-improvement Plan–Do–Study–Act model.	System II (TS by multidisciplinary breast specialists) had fewer inaccuracies than System I (TS by nonspecialist breast clinic staff) (33.78% vs. 51.67%, respectively; *p* = 0.05).
Nàpoles et al. (2019) [29]	America	Telehealth/Home visits	Nurses and MOs	Mixed-methods	Breast cancer	Evaluating the feasibility of an SCP for Spanish-speaking patients approaching the end of active treatment.	A total of 83% of women completed all 5 coaching calls. A total of 81% rated the quality of the app as “very good” or “excellent”.
Glaser et al. (2019) [32]	America	Cancer center	Oncological staff	Implementation projects	Breast and gynecologic cancer	Development of an SCP, a network of support services, and an integrative medicine program.	A total of 908 people accessed the survivorship clinic, receiving a complete clinical assessment and an SCP.
McGrath et al. (2019) [38]	America	Cancer center	Nurse teams	Quality-improvement projects	Different types of cancer	Standardizing how SCPs are integrated into the EHR.	Standardization of the SCP increased both the participation of the other specialists and increased the rate of completion from 10% to 34%.
Lee L.Y. et al. (2023) [39]	Asia	Cancer centers	Nurse	RCT	Head and neck cancer	Nurse-led SCP vs. usual care on physical/mental health emotional distress, social support, and resilience in 100 dyads.	In the IG, the endpoints enhanced after 6 months, with statistical significance.
Williamson- Butler et al. (2022) [40]	America	Hospital	Nurse	RCT	Breast cancer	POST (N = 100) vs. UC (N = 100) on patient ratings of quality and the content of discussion with providers at the end of their BC treatment.	The POST women endorsed 20 out of the 29 topics compared to 14 topics endorsed by the UC. The POST women reported a better QOD across all subscales.
Su et al. (2019) [41]	America	Tele-health	MO and PCP	RCT	Breast cancer	Web-based BC patient SCP (N = 61) vs. UC (N = 55) to improve on hot flashes, fertility-related concerns, contraception, and vaginal symptoms.	For the IG (70.9%) vs. UC (57.3%): fertility-related concerns (27.9% vs. 14.6%; OR 2.3); hot flashes (58.5% vs. 55.8%; OR 1.1); vaginal symptoms (42.5% vs. 40.7%; OR 1.1); contraception (50% vs. 42.6%; OR 1.4)

PCPs = primary care physicians; RCT = randomized controlled trial; CRC = colorectal cancer; SCP = survivorship care plan; VCSP = Victorian Cancer Survivorship Program; SC = survivor care; SCNs = supportive care needs; QOL = quality of life; CS = cancer survivorship; SCCP = survivorship care planning program; RAVs = risk-adapted visits; LIFE = Living in the Future; POST = Polaris Oncology Survivorship Transition; EHR = electronic health record; OCPs = oncology care providers; ACS = American Cancer Society; ASCO = American Society of Clinical Oncology; LLTE = late/long-term effect; TS = treatment summaries; EMR = electronic medical record; IG = intervention group; CG = control group; MO = medical oncologist; BCS = breast cancer survival; UC = usual care; QOD = quality of discussion; NA = not applicable.

## Appendix C

**Table A3:** TIDieR checklist analysis of SCP interventions from 2013 to 2018.

	Brief Name	Why	What Materials	What Procedures	Who Provided	How	Where	When and How Much	Tailoring	Modifications	How Well Planned	How Well Actual
Rosenberg et al. (2016) [21]	LIFE SCP	RAVs: (1) placing post-treatment CSs into the primary care setting; (2) diagnosis and treatments for CSs; (3) the active role of CSs in pursuing wellness; (4) linking CSs to community resources that will assist them in their recovery.	Face-to-face visit: 1 h for the provision and discussion of a personalized SCP, which was entered into the patient’s EPIC, EMR, and was also printed as a patient-friendly portable summary.	The LIFE entry point is a CS consulting RAV for those that have completed active treatment and are directed to the program within 1 year of the completion of medical treatment.	The LIFE program is directed by a physician; a certified oncology nurse is the clinical coordinator and conducts the RAV.	Referrals to the LIFE RAV: (1) sending an EPIC in-basket message with the patient’s chart attached to the LIFE clinical coordinator; (2) calling the LIFE line; (3) placing a CS outpatient order in EPIC.	Visits take place 5/7 days in any of the 3 hospital locations and depend on patient preference and provider availability as to the location, time, and date of the appointment.	LIFE participants are anonymously surveyed in two ways: immediately after their RAV and then at least 1 year after.				
Rosales et al. (2014) [22]	MSTI, with support from the NCCCP and ASCO	MSTI: the patient’s SCP is prepared in the EHR by a registered health information technician. This document is reviewed during an appointment with a nurse practitioner and social worker.	Medical record audit and follow-up telephone call.	When patients at the MSTI complete chemotherapy and/or radiation therapy, they are referred for a survivorship follow-up visit.	The visit lasts 1 h and consists of a joint visit with a nurse oncologist and oncological social worker of the MSTI clinic.	The nurse conducts an examination to the physical LLTE of the treatment, surveillance, and health promotion, discusses the importance of care coordination, and explains how this document will be shared with the PCP.	The social worker assists the nurse practitioner with the discussion of the SCP.	A total of 90 min of social work time, 75 min of nurse practitioner time, and 60 min of registered health information technician time.	The RHIT sends a copy of the patient’s treatment summary and care plan with the provider’s dictation to the PCP and referring physician.			
Jefford et al. (2015) [23]	VCSP	Pilot projects: (1) the post-treatment assistance of CSs in acute and primary care; (2) focus of the specific care needs of different groups of CSs; (3) assessing the VMS (effectiveness and transferability); (4) recommendations for better follow-up assistance.			Semistructured interviews were conducted with VCSP pilot project managers, clinicians, stakeholders, and family doctors. Motivational interviews of nurses (training/experience) were also conducted.		Telephone information and support lines are well-positioned to deliver SCP well-being coaching in an ongoing and economically sustainable way.		The VCSP has had difficulty involving MOs for the self-management of patients in SCPs; this may reflect the traditional follow- up, in which there is limited space for the integration of support for self-management.			
Dulko et al. (2013) [24]	JF	The SCPs used to survey oncology staff and PCPs regarding challenges of implementing SCPs were evaluated.	The JF packet was downloaded and given to each staff member, which was available to the APPs through the JF toolkit on the website.	Telephone interviews were conducted with patients about two months after the care plan visit.	The nurse is well positioned to create and deliver SCPs, transitioning patients from oncology care to a PCP in a shared-care model of optimal wellness.	The nurse presented and discussed the SCP with the CS at that appointment. Several days prior to the visit, patients at both sites received a telephone call to remind them of the upcoming appointment.		Accessing complete medical records is an obstacle to completing the SCPs. A 3–6-month window to develop and deliver the SCPs may be ideal.		SCP: (a) patient diagnosis and treatment, (b) follow-up care and secondary prevention, (c) information on the LLTEs of cancer treatments received, and (d) a list of national and local health promotion resources.		
Patt et al. (2013) [28]	IOM, COC	No universal model for CS delivery exists today, and program models vary significantly.		1° A survivorship visit 2–3 months after the initial therapy was complete, as this is a time when patients may be more receptive to these issues (1 h). There was also an additional 30–60 min in preparation for the midlevel provider before the visit to complete the initial SCP document.	In addition, there was a general assessment of the triage and referral needs for nutrition, exercise, physical therapy, counseling, and other services, as well as a decision to have the patient follow up for survivorship issues in 3–6 months for higher acuity issues, or 1 year if minimally active issues were identified.	The longitudinal model, however, disseminates the survivorship plan early on, but continues to follow the patient. Some survivorship programs are also integrated with long-term follow-up clinics and are merged with expected follow-up visits.	Texas Oncology Cancer Center, Austin.				Within community practice, the development of a survivorship program could be independent or in collaboration with a local hospital program.	The AYA Healthy Survivorship mobile app, with evidence-based self-assessment, a BMI calculator, children’s oncology group health links, resource links, a survivorship plan links; right, the ASCO’s Cancer Net tumor-specific mobile application showing some of the app’s educational content.
Downs-Holmes et al. (2014) [33]	Survivorship care, including the IOM, NCCN, ASCO, LiveStrong, and the ONS	Symptom assessment and collaboration of care in follow-up excelled; documentation supporting the other efforts in survivorship care were lacking. Survivorship education focused on community resources, diet, exercise, lymphedema, and recurrence.			An interdisciplinary team, include representation from the APRN, social workers, nurse navigators, and a survivorship coordinator or designated administrative support, as well as surgical, medical, and radiation oncologists.	ASCO guidelines for follow-up survivorship care with examinations every 3 months for the first 3 years, every 6–12 months for the 4th and 5th years, and annually thereafter. The goal of the program was to alternate visits among the BC team specialists to fulfill the guidelines set by the NCCN and ASCO for the coordination of care. The program has been successfully implemented, ensuring survivors that their providers are communicating in their ongoing care.				Many implications exist for nursing staff in an SCP, including the evaluation and documentation of distress, coping, fatigue, lymphedema, sleep disturbance, and menopausal symptoms. In addition, the reinforcement and encouragement of established patient goals for healthy lifestyle are crucial for change to occur. Education on symptom management, community, and hospital-based resources are also crucial.	Instrumental to the collaboration of care among providers and the documentation of the SCP is the responsibility of the nurse.	
Grant et al. (2015) [34]	Survivorship Program at CCO	Many BCSs continue to be seen by specialists for routine follow-up care, despite growing evidence that transitioning appropriate BCSs to primary care is safe and effective.	Software development and IT support.	Three main models of follow-up care were developed: (1) direct-to-primary care, (2) transition clinics, and (3) shared care.	The SCP was directed by a nurse, FHT, GPO, MO, and PCP.		Fourteen RCCs in Ontario, Canada.	An environmental scan after 1 year.	Analysis of the models described in the final reports submitted by each RCC identified three main models of follow-up care: (1) direct to the PCP, (2) the transition clinics, and (3) shared care.	All 14 RCCs developed an SCP, a transition letter, and patient education material. The SCP, in most cases, was populated by a nurse at the cancer center and was sent by fax or mail to the survivor’s PCP in the community. All SCPs included an up-to-date list of local resources for survivors.	CSs and their PCPs in these regions were offered direct access to a nurse via telephone. Transitioned survivors and their PCPs were provided direct access to a contact within the RCC who would be able to triage questions about follow-up care or recurrence.	
Berman et al. (2016) [35]	OncoLife and the LIVESTRONG (Internet-based)	Patients were asked about symptoms, disease characteristics, and previous and LLTEs. The characteristics of the PCF users were analyzed, and the PROs were related to the treatments provided.	Internet-based programs, publicly accessible via OncoLink, were created to design individualized SCPs for patients treated previously for cancer.		Nurses and physicians.			PROs have been shown to be prognostic for survival. Emerged patterns of longitudinal PROs were collected in the development of the SCP.				
Tevaarwerk et al. (2017) [36]		Treatment summaries prepared as part of the SCP should correctly and thoroughly report diagnosis and treatment information.	The EMR, external software program, and manual clinic record.	The EHR or using manual data entry into an external software program to create the summary.	A nurse reviewed each survivor’s medical records to abstract the necessary diagnosis and treatment data. The nurse then provided the document, typically as part of an SCP visit (either in person or telephone-based).		Two midwestern cancer centers.	As part of a clinical trial.				
Jefford et al. (2016) [42]	Nurse-led supportive care package for CRC	The improvement of the SCNs and QOL of patients with CRC. The intervention comprised of educational materials, needs assessments, SCPs, end-of-treatment sessions, and 3 follow-up telephone calls.	(a) Information package, (b) nurse-led, face-to-face end-of-treatment session; (c) a tailored SCP; (d) telephone follow-up.		Nurses received training in all aspects of the protocol into usual care.		Private hospital clinic room.	These sessions occurred 1, 3, and 7 weeks after the first intervention. The sessions revisited issues discussed during the end-of-treatment and addressed any other CS issues in 60 min.				

LIFE = Living in the Future; RAVs = risk-adapted visits; SCP = survivorship care plan; CS = cancer survival; EPIC = Epic Systems Corporation; EMR = electronic medical record; CSP = cancer survivorship program; MSTI = St. Luke’s Mountain States Tumor Institute; NCCCP = National Community Cancer Center Program; ASCO = American Society of Clinical Oncology; PPC = patient’s primary care; SMART = Specific, Measurable, Actionable, Realistic, Time-oriented; RHIT = registered health information technician; VCSP = Victorian Cancer Survivorship Program; GPs = general practitioners; GI = gastrointestinal; APPs = advanced practice professionals; NCCN = National Comprehensive Cancer Network; POST = Polaris Oncology Survivorship Transition; OCPs = oncology care providers; BC = breast cancer; LLTE = late/long-term effect; SCM = survivorship care model; PROs = patient-reported outcomes; DVD = digital versatile disc; QPL = question prompt list; PCPs = primary care physicians; TS = treatment summary; MELCARE = Survivorship Program for People with Metastatic Melanoma; MIA = Melanoma Institute of Australia; MPA = Melanoma Patients Australia; DT = distress thermometer; EHR = electronic health record; CRC = colorectal cancer; RCT = randomized controlled trial; SCNs = supportive care needs; QOL = quality of life; SC = survivor care; IOM = Institute of Medicine; RAs = research assistants; TAU = treatment as usual; DMGs = disease management groups; APRNs = advanced practice registered nurses; NCI = National Cancer Institute; SOP = standard operating procedure; SA = South Australia; SACS = SA Cancer Service; CP = care plan; M.R. = Director of Survivorship; COC = American College of Surgeons Commission On Cancer; US = United States; CCO = Cancer Care Ontario; IT = information technology; FHT = family health team; GPO = general practice oncologist; MO = medical oncologist; RCCs = regional cancer centers; DFCI = Dana–Farber Cancer Institute; ACS = American Cancer Society; LHE = lay health educator; CIS = The National Cancer Institute’s Cancer Information Services; ONS = Oncology Nursing Society; SP = survivorship program; Min = minutes; JF = journey forward; QOD = quality of discussion.

## Appendix D

**Table A4:** TIDieR checklist analysis of SCP interventions from 2019 to 2023.

	Brief Name	Why	What Materials	What Procedures	Who Provided	How	Where	When and How Much	Tailoring	Modifications	How Well Planned
Lai- Kwon et al. (2022) [25]	Nurse-led MELCARE		Telehealth-delivered: electronic survey after the follow-up consultation assessing the overall utility of MELCARE.		MELCARE was designed by a multidisciplinary team of healthcare professionals and consumers from the MIA and MPA. It consisted of two, 1 h, melanoma nurse-led consultations conducted via telephone 3 months apart.	All participants received MELCARE, a nurse-led survivorship program involving two telehealth consultations 3 months apart, a needs assessment using the DT and problem list, and the creation of an SCP.	Specialist melanoma center in Australia.	Administration of the DT and problem list in the initial nurse-led telehealth consultation of 60 min. Within 2 weeks of the initial consultation: produce an SCP (shared with participants, PCPs, and MOs). Three months after initial consultation: administer the DT and problem list follow-up in the nurse-led telehealth consultation of 60 min. After follow-up consultation: participants complete the utility survey.			
Lee et al. (2020) [26]	A bladder cancer SCP (ASCO, NCCN, IOM, and CoC)				A nurse and physicians.	Focus groups: 60 and 120 min with physicians (e.g., urologists and oncologists) and nonphysician providers (e.g., physician assistants, PAs, nurse practitioners, and social workers).	Twelve high academic health-centers in the US and Canada, and one private practice group enrolled patients in this prospective clinical pilot.	A mixed-method model in III phases; a 12.3 min SCP.			
Ivanics et al. (2019) [27]	Healthcare improvement’s Plan–Do–Study–Act model	System I involved TSs drafted by nonspecialist breast clinic staff; System II involved TSs vetted through a multidisciplinary breast specialist conference approach. The accuracy of the basic documentation entries related to dates and the components of the treatment were compared for the two approaches.	The EHR of these patients for the monitoring of the timeline regarding when each patient is due to receive the SCP–TS document.		The Breast Program Leadership Committee convenes monthly and includes representatives from surgery, MO, radiation, pathology, radiology, as well as nurses and physical therapy/rehabilitation medicine.	Breast oncology personnel or nurses.	A nurse and physician maintain an EHR of these patients for the monitoring of the timeline regarding when each patient is due to receive the SCP–TS document.				Patients requiring chemotherapy are assigned to have their SCP–TS drafted by a member of medical oncology; patients requiring surgery and no chemo/radiotherapy are assigned to a surgical team; patients receiving radiation as a component of their care, but no chemotherapy, are assigned to a radiation oncologist.
Nápoles et al. (2019) [29]	Mobile phone app “Nuevo Amanecer (New Dawn)” and telephone coaching for the SCP	Spanish-speaking Latina BCSs’ experience disparities in the knowledge of BC survivorship care, psychosocial health, lifestyle risk factors, and symptoms compared with their white counterparts. The SCP could help these women receive optimal follow-up care and manage their conditions.	Instructions on the use of the SCP; a booklet; the app installed; an unmasked activity tracker; an illustrated guide.		Completed SCPs were reviewed by the project director and the patient’s oncologist or oncology nurse, and scanned into the patient’s EHR.			A 2-month intervention. Home visit 1: a 45–60 min visit. Home visit 2: in this 1 h visit, participants received instructions.	The mobile app home page contained: daily walks, treatment, follow-up care, and managing symptoms.	Coaching consisted of 5 weekly phone calls with the following structure: daily steps goal and working through any barriers; 5 health topics: (1) walking and nutrition, (2) BC follow-up care, (3) signs of recurrence, (4) treatment LLTEs, and (5) resources and review of content from the first 4 calls.	Home visit 1: The RA conducted the visit at the clinic site or the participant’s home. Home visit 2: participants received materials and verbal instructions on the use of the written SCP. Home visit 3: At this visit, the RA conducted the final assessment and a brief satisfaction survey, synchronized the activity tracker, and collected the mobile phone and charger.
Corsini et al. (2020) [30]	SA CS framework	The framework was developed to identify and recommend the minimum level of care CSs should receive following the completion of treatment. Key components of the framework include the provision of a cancer TS and the development of a CP.	The NCCN DT and Problem checklist was utilized during individual consultations with the survivors to identify the key needs and priorities, and to establish goals to address these within the CP.	The time to TS and CP was 154 min (median 165 min) per person: for medical records, it was 20–90 min (median 50 min); for needs assessment, it was 45–90 min (median 60 min); for the delivery to survivors of the letter from GPs, it was 30–75 min (median 50 min).	Four self-selected teams consisting of a nurse candidate and an MO.	Face-to-face consultation with one nurse.	Four medical oncology clinics in South Australia participated (three metropolitan and one regional).	A 3-month pilot study; 165 min per person.			The CP template was revised to list resources at the bottom with reference to relevant websites. As a result of the early discussions at the debrief sessions, a list of key phrases and examples of common issues being identified within the CP were developed.
Fok et al. (2020) [31]	PCPs towards managing low-risk BCSs in a shared-care model with specialists	To explore the perspectives of PCPs towards managing BCSs in a community-based shared-care model.	For a shared-care model involving PCPs, an MO assumes the responsibility for cancer-related care, with the PCP focusing on the primary care. This model adopts a risk-stratified approach, where low-risk BCSs are managed by alternating visits with oncologists and PCPs.	Expanding PCPs’ role in survivorship care must go beyond relieving the MO to allow them to focus on active cancer treatment.		PCPs are best placed to address unmet needs in the psychosocial domains, optimize comorbidities, and ensure adherence to lifestyle modifications.	Singapore, which included private PCPs and public PCPs.	Qualitative study.	Recommendations from the PCP included risk stratification, role definition, focused training, timely communication, and sustainable funding to equip them for this expanded role. However, the successful implementation must be centered around instilling in the BCS the belief that her PCP is a valued partner in her cancer journey.		
Glaser et al. (2019) [32]	Survivorship program at Roswell Park Comprehensive Cancer Center (Roswell Park)	An SCP at an urban National Cancer Institute-designated comprehensive cancer center with three closely linked components: an SCP with a dedicated staff, a network of support services, including wellness, and an integrative medicine program.		(1) Patients’ complete treatment (one-time visit to survivorship provides the patient and family with a complete assessment at the start of the post-treatment period). (2) An avenue to survivorship is a transfer from oncology for long-term surveillance. (3) A way to access survivorship is through self-referral.	The SCP should not be solely contingent on the preferences of the oncology team.		Roswell Park Comprehensive Cancer Center (Buffalo, NY)	A 1-year pilot study.	These services would transfer patients to the centralized SCP, whereas others would prefer to provide an SCP within the oncology practices. The M.R. met with the other oncology disease sites to define a workable SCP pathway and to determine the optimal timeframe for transitioning these patients from the oncology service to the survivorship service.		
McGrath et al. (2019) [38]	LIVESTRONG, JF, and ASCO guidelines	(1) Develop an EHR; (2) evaluate an SCP within 3 to 6 months of the completion of therapy for survival visits; (3) develop site-specific treatment plans that meet ASCO standards; (4) pilot the implementation of an SCP using EMR functions; (5) assess the process.	Oncology DMGs created flow sheets that included information about CS treatments and LLTEs. These flowsheets and models are live documents for a disease-specific flow sheet.	The NP entered the data into the flow sheet, and this information was then easily uploaded into the hardened SCP document. Using the disease-specific flow sheet and the tempered SCP document, including the patient’s “smart text” education, it was validated that the completion of a treatment plan took less than 15 min for patients.	Research suggests that nurse survivorship clinics have been successful in providing quality SCPs in accordance with the IOM recommendations and demonstrate improvement in patient satisfaction, QOL, and process efficiency.	Nurse group evaluation: the audit was performed by a chart review using an EPIC/Beacon-generated list that utilized “curative intent” and the completion of a Beacon plan to create an eligible patient list. This list was then reviewed by the nurse group to assess the number of eligible patients who had completed the SCP.	NCI-designated academic medical center.	The timing of the delivery of the SCP was targeted between 3 and 6 months after completion of therapy to comply with the Commission on Cancer standards. Then, there was a 3- and 6-month evaluation of the project efficacy.		The SCP identifies medical oncology as the provider responsible for all combined modality patients. Radiation oncology is responsible for patients receiving definitive radiation therapy only.	The NP group responsible for delivering the treatment plans has met several times to reach a consensus on the “intelligent text” used in the model for the patient education. The “smart text” could be loaded into a patient’s SCP and easily edited for CSs.
Lee et al. (2023) [39]	A nurse-led SCP on the health and resilience of primary caregivers of patients with advanced head and neck cancer	Evaluate the effects of a nurse-led SCP on emotional distress, social support, physical health, mental health, and resilience in primary caregivers of patients with advanced head and neck cancer.			Five domains: (1) problems of primary caregivers; (2) cancer risk factors, the side-effects of treatment, caregiving burden, and possible contraindications; (3) caregiving skills and psychological supportive care; (4) health promotion and surveillance for cancer recurrence; (5) primary caregivers’ feedback on the effectiveness of the program.	The nurse with the dyads in the IG had monthly meetings for the first 6 months (1 h visit) to the clinic after the patient had completed the initial HNC treatment. Follow-up calls to discuss the problems and concerns of the primary caregivers were made twice a week. The CG received usual care in the health education room, including caregiving information regarding symptom management, daily care, and medical appointments.	A hospital in northern Taiwan.	A 4-year RCT with parallel, double-blind recruitment.			The nurse-led SCP can be applied before the patients complete the treatment, which may increase the positive effect on physical health and adaptation.
Williamson-Butler et al. (2022) [40]	POST	A web-based program: the EHR and from the MO providers generate an individualized SCP that abides by the IOM and ASCO guidelines.	SCP content checklist to examine the patient-reported quality and content of the discussion with their MO. Individualized SCP, which was created by the blinded for review RA and the study nurse, using both the patients’ EHR and their responses to the POST assessment.	The final SCP consisted of 10 patient-centered questions; every plan contained sections that included information on follow-ups, general recommendations for BCSs, possible LLTEs, screening and surveillance tips, etc. Finally, at the research visit, the TAU women received the affiliated hospital’s standard care planning procedure. Like the POST protocol, the TAU women also completed the final baseline assessments at this visit.	The nurse provides the POST to women ending treatment for BC: it may help women transition into survivorship feeling more knowledgeable about important survivorship topics and satisfied with their oncology care.	In person visit, follow-up via phone or e-mail at 1-, 3-, and 6-months postbaseline, which measured the psychological, physical, and emotional outcomes. On average, it took about 30 min for the study nurse to review the SCP with the POST women.	Last treatment visit in hospital oncology care.	The TAU women received an SCP. For these women, the nurse spent approximately 20 to 30 min reviewing the care plan with them.			
Su et al. (2019) [41]	BC SCP	BCSs who received a web-based, women’s health SCP were more likely to improve on at least one of four targeted issues compared to the attention controls.	(1) A 2-page SCP framed in a question-and-answer format; (2) a detailed summary of the systematic review results; (3) a description of the relevant clinical guidelines with hyperlinks to them; (4) curated web-based resources for CSs and providers.	The SCP was accessible to both survivors and the provider of their choice, as survivors actively seek health information from their providers and on the Internet.	MOs and PCPs.						

LIFE = Living In The Future; RAVs = risk-adapted visits; SCP = survivorship care plan; CS = cancer survival; EPIC = Epic Systems Corporation; EMR = electronic medical record; CSP = cancer survivorship program; MSTI = St. Luke’s Mountain States Tumor Institute; NCCCP = National Community Cancer Center Program; ASCO = American Society of Clinical Oncology; PPC = patient’s primary care; SMART = Specific, Measurable, Actionable, Realistic, Time-oriented; RHIT = registered health information technician; VCSP = Victorian Cancer Survivorship Program; GPs = general practitioners; GI = gastrointestinal; APPs = advanced practice professionals; NCCN = National Comprehensive Cancer Network; POST = Polaris Oncology Survivorship Transition; OCPs = oncology care providers; BC = breast cancer; LLTE = late/long-term effect; SCM = survivorship care model; PROs = patient-reported outcomes; DVD = digital versatile disc; QPL = question prompt list; PCPs = primary care physicians; TS = treatment summary; MELCARE = Survivorship Program for People with Metastatic Melanoma; MIA = Melanoma Institute of Australia; MPA = Melanoma Patients Australia; DT = distress thermometer; EHR = electronic health record; CRC = colorectal cancer; RCT = randomized controlled trial; SCNs = supportive care needs; QOL = quality of life; SC = survivor care; IOM = Institute of Medicine; RAs = research assistants; TAU = treatment as usual; DMGs = disease management groups; APRNs = advanced practice registered nurses; NCI = National Cancer Institute; SOP = standard operating procedure; SA = South Australia; SACS = SA Cancer Service; CP = care plan; M.R. = Director of Survivorship; COC = American College of Surgeons Commission On Cancer; US = United States; CCO = Cancer Care Ontario; IT = information technology; FHT = family health team; GPO = general practice oncologist; MO = medical oncologist; RCCs = regional cancer centers; DFCI = Dana–Farber Cancer Institute; ACS = American Cancer Society; LHE = lay health educator; CIS = National Cancer Institute’s Cancer Information Services; ONS = Oncology Nursing Society; SP = survivorship program; Min = minutes; JF = journey forward; QOD = quality of discussion.
